# Biofilm development by clinical isolates of *Staphylococcus* spp. from retrieved orthopedic prostheses

**DOI:** 10.3109/17453674.2010.537810

**Published:** 2010-11-26

**Authors:** Jaime Esteban, Diana Molina-Manso, Iris Spiliopoulou, José Cordero-Ampuero, Ricardo Fernández-Roblas, Antigoni Foka, Enrique Gómez-Barrena

**Affiliations:** ^1^Departments of Clinical Microbiology; ^2^Department of Microbiology, School of Medicine, University of Patras, Patras, Greece; ^3^Department of Orthopaedics, Hospital Universitario La Princesa, Madrid, Spain; ^4^Departments of Clinical Orthopaedics, Fundación Jiménez Díaz-UTE, Madrid

## Abstract

**Background:**

Biofilms are considered the key factor in the development of implant-related infections. However, only a few reports have dealt with the ability of organisms isolated from such infections to develop biofilms in vitro.

**Methods:**

We evaluated different phenotypic techniques (2 microtiter plate assays and confocal laser scanning microscopy (CLSM) and genotypic techniques (detection of the *ica* operon) related to biofilm development by clinical isolates of *Staphylococcus* spp.

**Results:**

All 26 strains tested (from 23 specimens) were biofilm producers. Stepanovic test detected biofilm formation in 85% of the strains, microtiter plate assay in 65%, and CLSM in 39%. The ***ica*** operon was detected in 73% of all strains (all 13 *S. aureus* strains and 6 of the 13 coagulase-negative *Staphylococcus* strains). 7 *ica*-negative strains were biofilm-positive by phenotypic methods.

**Interpretation:**

The detection of ***ica*** genes could not be related to the phenotypic ability of the strains to develop a biofilm in vitro, so both studies (genetic and phenotypic) are required for a better evaluation of the biofilm-producing ability of clinical strains of *Staphylococcus* isolated from orthopedic infections.

Prosthetic joint infection is an emerging problem due to the increase in surgical techniques that involve the use of biomaterials. It has been estimated that infection rates of orthopedic prostheses of the hip and knee range from 1% to 2%, but they can be as high as 9% for elbow prostheses (Darouiche 2004). The main pathogenic factor for the development of the infection is the ability of bacteria to form a biofilm. Biofilm has been defined as a multicellular community composed of prokaryotic and/or eukaryotic cells embedded in a matrix composed, at least partially, of material synthesized by the sessile cells in the community (Costerton 2007). Biofilm development has important consequences for the management of the patient: sessile bacteria become antibiotic-resistant, and treatment is therefore more complicated than treatment of acute bacterial infections where biofilms are not involved (Patel 2005). Moreover, bacteria in biofilms are more difficult to isolate, so the diagnosis of these infections requires techniques different from those used in conventional microbiology laboratories (Trampuz et al. 2007, Esteban et al. 2008). The main extracellular substance involved when staphylococci are embedded in biofilms, which has received much attention, is the polysaccharide intercellular adhesin (PIA) (Cafiso et al. 2004, Gara 2007). PIA is encoded by the genes of the *ica* operon (*icaA, icaD, icaB, and icaC*), which are negatively regulated by *icaR* (Arciola et al. 2002, Cafiso et al. 2004, Gara 2007).

Many studies on biofilm formation by clinically relevant bacteria have been performed. However, there is limited information available from studies performed on clinical isolates from prosthetic infections where several techniques have been used to evaluate biofilm formation. Here we report a study comparing phenotypic and genotypic techniques that have been developed to study biofilm formation. These were applied to clinically relevant strains of *Staphylococcus* spp.

## Material and methods

### Bacterial strains

26 clinical isolates of *Staphylococcus* spp. were included in the study. The strains were isolated from retrieved orthopedic prostheses from 22 consecutive patients with staphylococcal deep prosthetic joint infections at two major university hospitals (Fundación Jiménez Díaz and La Princesa, Madrid, Spain). All the patients were diagnosed as having prosthetic joint infection according to clinical criteria (Cordero-Ampuero et al. 2007).

The strains were isolated using a sonication protocol previously described (Esteban et al. 2008). The isolates were identified by conventional biochemical tests (coagulase) and commercial identification galleries (API-STAPH; bioMérieux, Marcy L'Etoile, France) and were maintained frozen at –20ºC in skimmed milk until experiments were performed. *S. epidermidis* ATCC strain 35984 (a biofilm-producing strain) and *S. aureus* strain 15981, kindly provided by Dr. Lasa (Valle et al. 2003), were used as biofilm-positive control strains. Sterile PBS was used as negative control.

The informed consent in our hospital includes authorization to handle samples from the patients, including implants obtained during surgery. No other approval by our Institutional Review Board was necessary because only bacterial isolates were analyzed, and neither samples nor patients were studied in this work.

### Biofilm formation studied by microtiter plate assay

We performed the microtiter plate assay (MP) described by Christensen et al. (1985), with modifications. A suspension equivalent to the McFarland 0.5 turbidity standard was prepared in Müller-Hinton broth (Becton Dickinson, Franklin Lakes, NJ) for each strain. Accuracy of bacterial counts in the suspension was confirmed by serial dilution in log steps. 100 μL from each bacterial suspension was inoculated onto 96-well Costar tissue culture microtiter plates (Corning). These were incubated at 37ºC for 24 h in a normal atmosphere. After incubation, the medium was removed and replaced with 100 μL sterile Müller-Hinton broth. Samples were incubated for another 24 h under the same conditions to obtain enough growth from strain P-95, which showed slow growth characteristics and almost no growth after only 24 h of incubation. The medium was then removed, and the wells were washed 3 times with sterile distilled water. 150 μL of crystal violet (Panreac, Barcelona, Spain) was added to each well and left for 45 min at room temperature. The dye was then removed, and this was followed by 5 washings with sterile distilled water. The preparations were then destained with 200 μL of 95% ethanol for 3 min. Finally, 100 μL of colored ethanol from each sample was transferred to another microtiter plate. The optical density (OD) of the ethanol-dye suspension was measured at 540 nm with an Opsys MR spectrophotometer (Dynex Technologies, Richfield, MN). The ODs obtained were compared with those of negative controls (wells without bacterial inoculum). We considered the ODs 0.150 to be negative (mean value of all negative controls included in the tests), and those with OD > 0.150 to be positive. All the strains were tested in triplicate in 3 experiments, and the average value for each sample was calculated.

We also used the Stepanovic method as a control for our protocol, using the same medium. This was also repeated twice and all the strains were tested in triplicate. Evaluation of the results was performed using the scale described by Stepanovic et al. (2007).

### Confocal laser scanning microscopy (CLSM)

A McFarland 0.5 standard suspension was prepared for each strain in Müller-Hinton broth. 6-for-4 microtiter plates (Nunc, Roskilde, Denmark) with a sterile Thermanox disk for each well were inoculated with 1 mL of each suspension. The plates were incubated for 24 h at 37ºC. The medium was then changed using 1 mL of Müller-Hinton broth and the preparations were re-incubated for another 24 h. After incubation, the medium was removed and the wells were washed 3 times with sterile distilled water. The disks were then removed and stained with Live/Dead BacLight stain (Invitrogen), using the instructions provided by the manufacturer. Stained disks were examined with a Leica DM IRB confocal laser scanning microscope at 40× magnification. The experiment was performed in triplicate and repeated 2–3 times for all the strains. Several photographs were taken for all strains. The percentage of covered surface was calculated using ImageJ software for live and dead bacteria separately, and then the result was used to calculate the proportion of both types in the biofilm.

### Detection of the ica gene

After isolation of chromosomal DNA from the strains studied, amplification of *icaR* and the 4 genes of the *ica* operon was performed by PCR with specific primers (Table 1), according to previously described protocols (Cafiso et al. 2004, Gara 2007). *S. epidermidis* strain ATCC 35984 (RP62A), a biofilm-forming strain, and a biofilm-negative one (ATCC 12228) were used as positive and negative controls, respectively (Christensen et al. 1982a, b). The PCR products were analyzed by agarose gel electrophoresis.

**Table 1.** Sequences of the primers used, sizes of the expected PCR products, and references

Gene	Primer	Sequence (5'-3')	PCR product (bp)	Reference strain	Reference
*icaA*	icaA-F	TCTCTTGCAGGAGCAATCAA		ATCC35984	Arciola et al. (2002)
	icaA-R	TCAGGCACTAACATCCAGCA	188		
*icaD*	icaD-F	ATGGTCAAGCCCAGACAGAG		ATCC35984	Arciola et al. (2002)
	icaD-R	CGTGTTTTCAACATTTAATGCAA	198		
*icaB*	icaB-F	ATGGCTTAAAGCACACGACGC		ATCC35984	Cafiso et al. (2004)
	icaB-R	TATCGGCATCTGGTGTGACAG	526		
*icaC*	icaC-F	ATCATCGTGACACACTTACTAACG		ATCC35984	Cafiso et al. (2004)
	icaC-R	CTCTCTTAACATCATTCCGACGCC	934		
*icaR*	icaR-F	TACTGTCCTCAATAATCCCGAA		ATCC35984	Cafiso et al. (2004)
	icaR-R	GGTACGATGGTACTACACTTGATG	453		

### Statistics

Statistical analysis was performed for *S. aureus* and *S. epidermidis* isolates, and in some cases between *S. aureus* and all other coagulase-negative staphylococci. Comparison between sensitivity of tests and species was performed using Chi-square and Fisher's exact tests. The calculations were performed using EPI-INFO software version 3.5.1 (Centers for Disease Control and Prevention, Atlanta, GA).

## Results

Of the 26 strains included in the study, isolated from 23 clinical specimens (22 patients), 13 were *Staphylococcus aureus* and 13 were coagulase-negative staphyloccoci (CoNS) (10 *S. epidermidis*, 1 *S. hominis*, 1 *S. warneri*, and 1 *S. lugdunensis*). 13 of the strains had been isolated from clinical specimens where more than one bacterium was recovered (polymicrobial infections), some of them including other bacterial genera (data not shown).

All 26 strains were positive for biofilm development (Table 2). The overall sensitivity of the Stepanovic method (STEP) was 89%, that of MP was 65%, and that of CLSM was 39%. For *S. aureus* isolates, the sensitivity of the techniques was as follows: STEP 85%, MP 62%, and CLSM 39%. For *S. epidermidis* isolates, the results were: STEP 90%, MP 70%, and CLSM 50%.

**Table 2.** Results obtained with each technique

Patient no.	Sample no.	Strain	Species	Isolated in conventional culture	MP/OD (SD) **^a^**	STEP **^b^**	CLSM (%) **^c^**	*ica***^d^**	*icaR*
1	1	P-1	*S. aureus*	Yes	0.232 (0.071)	1+	+ (49.9)	+	+
2	2	P-2	*S. aureus*	Yes	0.139 (0.040)	1+	+ (35.2)	+	–
6	4	P-4	*S. aureus*	No	0.196 (0.065)	2+	-	+	–
10	18	P-18	*S. aureus*	Yes	0.128 (0.043)	1+	+ (58.1)	+	–
11	19	P-19	*S. aureus*	No	0.134 (0.034)	1+	–	+	–
20	38	P-38	*S. aureus*	No	0.153 (0.048)	1+	+ (43.7)	+	–
45	41	P-41	*S. aureus*	Yes	0.122 (0.024)	1+	–	+	–
30	61	P-61.3	*S. aureus*	Yes	0.172 (0.043)	0+	–	+	–
30	61	P-61.4	*S. aureus*	Yes	0.151 (0.040)	1+	–	+	–
30	62	P-62.1	*S. aureus*	Yes	0.161 (0.054)	1+	+ (77.1)	+	–
33	68	P-68	*S. aureus*	Yes	0.155 (0.041)	1+	–	+	–
39	82	P-82.1	*S. aureus*	Yes	0.160 (0.056)	1+	–	+	–
49	95	P-95	*S. aureus*	Yes	1.109 (0.361)	0+	–	+	+
4	6	P-6.2	*S. epidermidis*	No	0.171 (0.122)	1+	–	+	–
4	6	P-6.5	*S. epidermidis*	No	0.234 (0.052)	3+	+ (51.2)	+	–
12	23	P-23.2	*S. epidermidis*	Yes	0.136 (0.024)	1+	–	–	–
18	33	P-33.1	*S. epidermidis*	Yes	0.256 (0.054)	3+	+ (54.6)	–	–
26	53	P-53.2	*S. epidermidis*	Yes	0.206 (0.090)	2+	+ (50.0)	+	+
27	55	P-55	*S. epidermidis*	No	0.209 (0.031)	1+	+ (51.6)	+	+
30	61	P-61.1	*S. epidermidis*	No	0.146 (0.027)	1+	–	–	–
30	61	P-61.2	*S. epidermidis*	No	0.139 (0.040)	1+	–	–	–
35	74	P-74	*S. epidermidis*	Yes	0.297 (0.052)	1+	–	+	+
52	101	P-101	*S. epidermidis*	Yes	0.236 (0.138)	0+	+ (49.7)	–	–
12	23	P-23.1	*S. warneri*	No	0.160 (0.035)	1+	–	–	–
26	53	P-53.7	*S. hominis*	No	0.219 (0.069)	2+	–	+	+
31	65	P-65	*S. lugdunensis*	Yes	0.139 (0.033)	1+	–	–	–

**^a^** MP/OD: microtiter plate assay / mean optical density (SD);

**^b^** STEP: Stepanovic test: 0+ (no biofilm), 1+: weak biofilm producer, 2+: moderate biofilm producer, 3+: strong biofilm producer;

**^c^** CLSM: confocal laser scanning microscopy assay (percentage of live bacteria inside the biofilm).

**^d^** *ica*: PCR detection of *icaA* and/or *icaD* genes.

**Figure 1.**
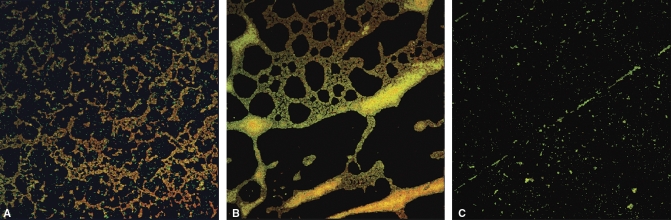
CLSM images. A. Biofilm-positive *S. aureus* (strain P-1). B. Biofilm-positive *S. epidermidis* (strain P-6.5). C. Biofilm-negative *S. aureus* (strain P-41).

Although CLSM had the lowest percentage of positive results, it allowed us to study the percentage of live and dead bacteria (Table 2). Among the CLSM-positive strains, the mean percentage of covered surface was 52 (SD 11, range 77–44). CLSM images showed either mature biofilm (with the strains considered biofilm-positive) or isolated bacteria without any biofilm development (the biofilm-negative strains) (Figure 1). The mean percentage of covered surface was 43% (SD 18, range 21–67).

**Figure 2.**
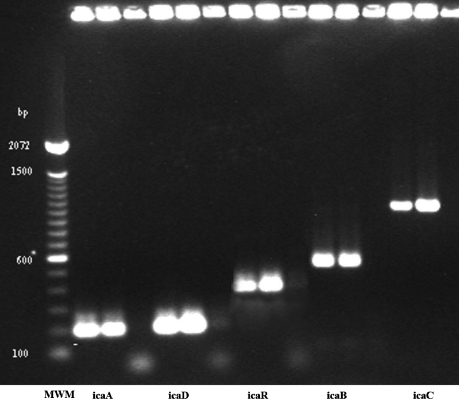
Agarose gel electrophoresis of PCR products. Each PCR run included the reference ATCC 35984 *ica*-positive strain, and a positive and a negative strain from our collection.

19 of the 26 strains were *ica*-positive, including all 13 of the *S. aureus* strains, half of the *S. epidermidis* strains, and altogether 6 of the 13 CoNS. A statistically significant difference for *ica* detection was detected between *S. aureus* and *S. epidermidis* (p = 0.007, Fisher's exact test) (Figure 2). This difference was also found to be significant when all CoNS strains were included in the comparison (p = 0.002, Fisher's exact test).

6 *ica*-positive strains also carried *icaR* (2 *S. aureus* and 4 CoNS), and among them, 3 were biofilm-positive by both phenotypic methods (Table 1). No statistically significant difference was found in detection of *ica R* between these groups. 7 *ica*-negative CoNS strains (5 *S. epidermidis,* 1 *S. lugdunensis,* and 1 *S. warneri*) were biofilm-positive.

There were no statistically significant differences between *S. aureus* and *S. epidermidis* in the results of phenotypic tests.

## Discussion

Biofilms have been increasingly implicated as a key pathogenic factor in biomaterial infections (Costerton 2005, 2007). Recently, new diagnostic methods have been designed based on the characteristics of biofilms, with better results than the classical methods of assessment (Trampuz et al. 2007, Esteban et al. 2008). To evaluate phenotypic and genotypic methods that can be used in the study of clinically relevant staphylococci that can produce biofilms, we performed this study which shows that different methods are required to fully detect biofilm-forming potential in isolates.

Of the different techniques used to evaluate the ability of different bacterial strains to produce biofilms, the crystal violet microplate assay technique—initially described by Christensen et al. (1985)—is one of the most popular. Based on the property of biofilms of retaining the dye despite washing with water, destaining with ethanol allows quantification of the amount of biofilm if one measures the amount of dye present in the ethanol by spectrophotometry. The usefulness of this technique has been evaluated using different organisms, and became popular because of its simplicity. A recent study using *S. epidermidis* isolated from orthopedic samples showed a higher number of positive results than molecular biology techniques (Arciola et al. 2006). This discrepancy may be due to the different sequences selected as targets for the molecular detection, because several genes are involved in biofilm development, and primer selection cannot detect all possibe genes involved.

The Stepanovic method is another technique with similar principles, although more standardized than the Christensen method. Here we found some discrepancies between these two techniques. The most notorious was an *S. aureus* strain that had the highest OD readings by the modified Christensen test but which appeared to be negative with the Stepanovic test (strain P-95). This particular strain had a lower growth rate than other strains, with minute colonies after 24 h at 37ºC, and it was also negative by the CLSM technique. Our modification of the Christensen method included a longer incubation period; this could be an explanation for this discrepancy, because biofilm development takes more time for this strain. This is a very important issue, since it is reported that *S. aureus* strains involved in osteoarticular infections (especially small-colony variants, or SCVs) are slowly growing strains (von Eiff 2008), and biofilm detection probably requires longer periods of time than those used for common strains.

CLSM has become one of the reference techniques for the study of biofilms (Donlan 2001, Lawrence et al. 1991). This technique allows analysis of the internal structure of biofilms through the use of microscopy combined with fluorescent stains, such as Backlight Live-Dead stain (Boulos et al. 1999). Despite this, no studies have been published concerning its usefulness as a screening technique for a broad number of clinical strains of *Staphylococcus* spp. In our study, CLSM detected a lower number of biofilm-producing strains than the crystal violet microtiter plate assay. This difference was more evident for CoNS than for *S. aureus*, where the number of biofilm-positive strains was the same with both techniques. This could be due to technical problems, which can be attributed to the thermanox slides; these may have different adherence properties than other polymers. However, CLSM allowed us to determine the percentage of live/dead bacteria in our isolates, so we believe that this technique is still useful for the study of more detailed aspects of biofilms, although selection of the substrate appears to be important for the study of particular strains, because of the observed differences between them. However, because of its low sensitivity, the method cannot be used to evaluate the ability of different strains to produce biofilms in a screening study.

Molecular detection of *ica* genes revealed the presence of the operon in most clinical strains (19 of 26). In addition, of the 6 *ica*-positive strains carrying *icaR*, 3 were negative for biofilm formation only by one of the phenotypic methods used. This indicates that carriage of *icaR* in not always related to inactivation of the *ica* operon, as reported elsewhere (Cafiso et al. 2004). However, 7 CoNS strains were biofilm-positive, even though they did not carry the *ica* operon. This has been described previously, and can be explained by the presence of other genetic markers (Fitzpatrick et al. 2005). This suggests the existence of ica-independent mechanisms of biofilm formation (Gara 2007).

In conclusion, all strains tested in our study could produce biofilm that could be detected with at least one of the techniques used. According to our results, molecular detection of the *ica* operon is associated with biofilm production. However, the absence of *ica* genes did not exclude the phenotypic ability of the strains to develop biofilm in vitro, so both approaches (genetic and phenotypic) are required for optimum evaluation of the biofilm producing ability of clinical strains of *Staphylococcus* isolated from orthopedic infections.
